# Temporal trends in genetic data and effective population size support efficacy of management practices in critically endangered dusky gopher frogs (*Lithobates sevosus*)

**DOI:** 10.1002/ece3.2084

**Published:** 2016-03-16

**Authors:** Kristin M. Hinkson, Stephen C. Richter

**Affiliations:** ^1^ Department of Biological SciencesEastern Kentucky UniversityRichmondKentucky40475; ^2^ Division of Natural AreasEastern Kentucky UniversityRichmondKentucky40475

**Keywords:** Conservation genetics, effective size, endangered species, genetic compensation, genetic structure, temporal variation

## Abstract

Monitoring temporal changes in population genetic diversity and effective population size can provide vital information on future viability. The dusky gopher frog, *Lithobates sevosus*, is a critically endangered species found only in coastal Mississippi, with low genetic variability as a consequence of isolation and population size reduction. Conservation management practices have been implemented, but their efficacy has not been addressed. We genotyped individuals collected 1997–2014 to determine temporal trends in population genetic variation, structure, and effective size. Observed and expected heterozygosity and allelic richness revealed temporally stable, but low, levels of genetic variation. Positive levels of inbreeding were found in each year. There was weak genetic structure among years, which can be attributed to increased effects of genetic drift and inbreeding in small populations. *L. sevosus* exhibited an increase in effective population size, and currently has an estimated effective size of 33.0–58.6 individuals, which is approximately half the census size. This large ratio could possibly be explained by genetic compensation. We found that management practices have been effective at maintaining and improving effective size and genetic diversity, but that additional strategies need to be implemented to enhance viability of the species.

## Introduction

The maintenance of genetic variation within populations and species is a primary concern in conservation biology because this variation allows species and populations to adapt to their changing environments (Frankham et al. 2004). Without this adaptive potential, populations are at an increased risk of endangerment and extinction. Habitat loss and fragmentation are major threats to genetic variation because they result in small, isolated populations (Keller et al. 2004; Richter et al. 2009; Smith et al. 2009). Ultimately, the persistence of these populations depends on the retention of genetic diversity. However, small, isolated populations have lower amounts of genetic variation and are more prone to inbreeding depression (Frankham 1996; Richter and Nunziata 2014). Species residing in wetland habitats, especially amphibians, are particularly vulnerable because in addition to relying on both wetland and upland habitats, most of the natural wetlands crucial to amphibian breeding events have been destroyed or degraded, causing a reduction in habitat and wetland species' population sizes (Dahl 2000; Beebee 2005; Curado et al. 2011).

Assessing temporal change in genetic variation of populations provides both historic and contemporary information and can reveal previous population bottlenecks, effects of genetic drift, and whether genetic variation is increasing or decreasing within a population (Heath et al. 2002; Bouzat 2010; Cuveliers et al. 2011). Many populations have decreased in genetic diversity over time due to anthropogenic and ecological factors (Arnaud and Laval 2004; Gomaa et al. 2011). Therefore, monitoring population genetic variation over time can serve as a necessary and valuable indicator of extinction risk and efficacy of current conservation management practices.

An informative parameter in evaluating maintenance of genetic variation both temporally or at a single point in time is effective population size (*N*_e_), which can be estimated through the use of microsatellite markers. Effective population size is defined as the size of the ideal population that experiences genetic drift at the same rate as the population of interest (Wright 1931). More generally, effective population size is the number of reproductively successful and genetically contributing individuals in a population. Effective population size provides insight to the amount of genetic variation being retained within a population, as *N*_e_ is typically inversely related to amount of inbreeding, especially in isolated populations, and to loss of genetic diversity (Beebee 2009; Hoehn et al. 2012). These estimates are crucial to conservation managers because they offer information on the genetic viability of a population, and they can serve as the basis for predictions on future viability (Hare et al. 2010).

Effective population size estimates and census sizes (*N*_c_) can be used together to calculate a *N*_e_/*N*_c_ ratio. This ratio is useful because census size is easier to measure than effective population size (Ardren and Kapuscinski 2003). Therefore, if a typical ratio could be determined, then it could be applied to populations where only census size is known, allowing for the simple estimation of *N*_e_, thus predicting rate of genetic loss (Ardren and Kapuscinski 2003). This ratio is actively pursued in research (Palstra and Ruzzante 2008; Luikart et al. 2010); however, no consensus has been reached on what constitutes a typical range of *N*_e_/*N*_c_ for natural populations, and it is questionable if such a range exists (Nunney 1993; Scribner et al. 1997; Hoffman et al. 2004). Additionally, research has shown that variance in reproductive success and family size can have large effects on this ratio (Serbezov et al. 2012). However, the temporal stability of *N*_e_/*N*_c_ estimates in natural populations remains an understudied area, and there is a need for improved understanding of this ratio (Ardren and Kapuscinski 2003; Luikart et al. 2010; Cuveliers et al. 2011; Jansson et al. 2012).

Many effective population size estimators share common assumptions (i.e., the population is sampled at random, there is no immigration, there is no selection, and there are no overlapping generations) (Waples 1989; Berthier et al. 2002; Tallmon et al. 2008; Waples and Do 2008). Of these assumptions, the need for discrete generations is often violated in natural populations, specifically in anurans (Beebee 2009). Research has shown that ignoring this assumption and proceeding with *N*_e_ estimates can lead to biased results because changes in allelic frequencies are not only solely due to genetic drift but also to age‐specific birth and survival rates (Jorde and Ryman 1995; Waples and Yokota 2007; Robinson and Moyer 2012). To circumvent this assumption, researchers have found procedures that minimize biases associated with overlapping generations. Waples and Yokota (2007) found by separating temporal samples 5–10 years apart, biases largely disappear, and Scribner et al. (1997) suggested that a population with high adult mortality is largely equivalent to discrete generations. For the purposes of this study, a combination of low adult survivorship and low rate of return after the first breeding event help to eliminate the biases associated with this assumption violation (Scribner et al. 1997; Richter and Seigel 2002). Further, while cognizant of possible limitations, few species have discrete generations, and these models have been widely applied to systems with overlapping generations (Scribner et al. 1997; Robinson and Moyer 2012; Hoffman et al. 2004; Waples 2005). Additionally, it is important to distinguish that for species with overlapping generations, such as anurans, single‐sample effective population size estimates most directly reflect the effective number of breeders for one reproductive season (*N*_b_), as opposed to *N*_e_ (Waples 2005). The relationship between *N*_b_ and *N*_e_ can be complex, but in many cases *N*_b_ serve as comparable measure to *N*_e_ (Waples 2005).

The dusky gopher frog (*Lithobates sevosus*) is a prime study species in determining the effects of population isolation on genetic diversity, population structure, and effective population size and in evaluating the efficacy of management practices. The species is listed as Endangered by the United States Fish and Wildlife Service (2001) and Critically Endangered by the International Union for Conservation of Nature and Natural Resources (2008). First described by Goin and Netting (1940), the historic geographic range of *L. sevosus* occurred from the coastal plains of Louisiana, Mississippi, and Alabama west of the Mobile Basin, primarily in longleaf pine (*Pinus palustris*) forests (Richter and Jensen 2005).

Now extirpated from the majority of its historical range, *L. sevosus* is only known to exist in two populations in coastal Mississippi, separated by 32 km. The population at Glen's Pond in Harrison County has a census population size estimate of 100–200 individuals, while the population in Mike's Pond in Jackson County (discovered in 2004) appears to have fewer than 20 adults and could possibly be extinct based on egg counts (Richter et al. 2001, 2009). Additionally, in 2013, some individuals dispersed to Pony Ranch Pond—a restored pond 1.3 km from Glen's Pond—and natural breeding occurred in both 2013 and 2014 (Pechmann and Tupy 2013; J. Tupy, pers. comm.). Previous research documented a maximum distance travelled of 299 m (Richter et al. 2001); however, the movement to Pony Ranch Pond indicates a greater dispersal capability.

Extensive genetic and demographic research has been conducted (Richter et al. 2001, 2003, 2009; Richter and Seigel 2002; Richter and Nunziata 2014), and many conservation management practices have been suggested and implemented (Richter and Seigel 1996, 1997, 1998; Seigel and Kennedy 2000; Seigel et al. 2001; Thurgate et al. 2002; Sisson 2003, 2004, 2005; Baxley and Qualls 2006, 2007; Sisson et al. 2008; Lee 2011). Specifically, a headstarting program (i.e., rearing tadpoles in cattle tanks and releasing them at a late larval stage or postmetamorphosis) was initiated in 2002, which represents the midpoint of our study. Other management practices include habitat management (i.e., prescribed fires) and restoration/creation of nearby wetlands. However, despite expansive research and management, studies have yet to address how inbreeding, genetic drift, and lack of gene flow caused by population isolation have affected changes in genetic variability of *L. sevosus* over the past 18 years, or the relative influence of implemented conservation management practices. Therefore, our objectives were to (1) determine temporal trends in population genetic variation and implications for future population persistence for *L. sevosus* based on these data and (2) estimate effective population size (or effective number of breeders). This information will allow for an evaluation of the effectiveness of current management practices in terms of preserving and enhancing genetic variability. Furthermore, this study hopes to inform broadly applicable questions in the field of conservation biology, including (1) Is there temporal genetic structuring in isolated populations? (2) How do conservation management practices, like tadpole headstarting, impact genetic diversity over time? and (3) Are *N*_e_/*N* ratios stable overtime in small, isolated populations?

The presence of extensive demographic data spanning the entirety of this study makes this research particularly applied and unique. Genetic studies rarely have the ability to use long‐term demographic data to bolster long‐term genetic data, and vice versa. Through our objectives, we intend to address how an endangered species—with a documented decline in reproductive output and previous population bottlenecks—has fared prior to and following implementation of conservation management practices in terms of genetic diversity, inbreeding, and effective size.

## Materials and Methods

### Sample collection

*Lithobates sevosus* egg samples were collected between 1997 and 2014 from individuals at Glen's Pond, which is located in De Soto National Forest (Harrison County, Mississippi). This population was selected because Glen's Pond represents the largest, if not only remaining, population and has the most stable breeding activity. Specifically, one egg was collected from each clutch deposited in 1997 (*n* = 58), 2005 (*n* = 20), 2008 (*n* = 50), 2013 (*n* = 31), and 2014 (*n* = 51). These years were selected to address the efficacy of the headstarting program, with 1997–2005 largely representing preconservation management practices, and 2006–2014 representing postconservation management practices. Individuals were genotyped for nine previously developed microsatellite loci, four loci from Richter and Broughton (2005) and five loci from Nunziata et al. (2012). DNA was extracted using the Qiagen DNeasy Blood and Tissue protocol (Qiagen, Inc., Valencia, CA). All individuals were genotyped for the nine microsatellite loci via PCR. See Richter and Broughton (2005) and Nunziata et al. (2012) for details about PCR conditions and characteristics of each locus (e.g., repeat motif and number of alleles per locus). The resulting PCR products were genotyped using an ABI 3100 Genetic Analyzer (Applied Biosystems, Inc., Foster City, CA). Allele lengths were scored using Gene Mapper version 4.0 (Applied Biosystems, Inc.).

### Statistical analyses

Tests between all pairs of loci were assessed for linkage disequilibrium, and deviations from Hardy–Weinberg equilibrium were assessed per locus per sample at the 5% nominal level with Bonferroni corrections using FSTAT Version 2.9.3.2 (Goudet 1995). The computer program Micro‐Checker was used to test for null alleles (Van Oosterhout et al. 2004). To determine the amount of genetic variation within *L. sevosus* for each year*,* observed (*H*_o_) and expected heterozygosity (*H*_e_) were calculated using GenAlEx Version 6.501, and allelic richness using rarefaction were calculated using FSTAT Version 2.9.3.2 (Goudet 1995; Peakall and Smouse 2006, 2012). Wright's inbreeding coefficient (*F*_IS_) was calculated using GENETIX 4.05 with 95% CIs generated via bootstrapping 10,000 times within each year (Belkhir et al. 2004). Friedman's tests were run to determine whether significant differences in *H*_o_, *H*_e_, allelic richness, and *F*_IS_ existed between the sample years. When global tests were significant, Wilcoxon signed‐rank tests for related samples were used to test pairwise differences between years. Overall and pairwise *F*_ST_ comparisons were calculated to determine whether significant genetic differences existed temporally using FSTAT Version 2.9.3.2, with 95% confidence intervals calculated for the overall value via bootstrapping over loci (Weir and Cockerham 1984; Goudet 1995) and pairwise significance values calculated with 10,000 permutations using Arlequin Version 3.5.2.1 (Excoffier and Lischer 2010). A regression of *F*_ST_/(1‐*F*_ST_) on temporal distance was computed, and a Mantel test was performed using FSTAT Version 2.9.3.2 to assess significance levels (Goudet 1995). Mantel tests investigate correlations between two matrices, with one matrix usually as geographic distance. However, researchers have also used temporal distance in lieu of geographic distance because the mathematical and biological relationship is similar to comparisons with distance (Bernal‐Ramírez et al. 2003; Haber et al. 2005).

Additionally, to assess the impact of pre‐ and postconservation efforts (i.e., farm‐reared tadpole supplementation) on heterozygosity, allelic richness, and inbreeding, a two‐way repeated measures ANOVA was performed such that year 1997 represents “early” preconservation efforts, year 2005 represents “late” preconservation efforts, year 2008 represents “early” postconservation efforts, and year 2014 represents “late” postconservation efforts. Year 2013 was omitted from these analyses to create a 2 × 2 level factor design.

### *N*_b_ and *N*_e_ estimates

Two methods to estimate effective size were used—a single‐sample estimator and a temporal estimator. The single‐sample estimate was calculated with Bayesian partial likelihood analysis using ONeSAMP 1.2 (Tallmon et al. 2008). Specified upper and lower *N*_b_ limits were set to 4 > *N*_b_ > 500. The *N*_b_ of simulated populations with summary statistics resembling that of the test population were used to infer *N*_b_ of the target population (Tallmon et al. 2008). Effective number of breeders was calculated for years 1997, 2005, and 2014 to represent effective sizes pre‐ and postfarm‐reared tadpole supplementation.

The temporal estimate of *N*_e_ was found using a pseudolikelihood method (Wang 2001). Calculations for the pseudolikelihood method were computed using the computer program MNE 1.0 (Wang 2001). This model assumes that populations are closed and that allele frequency changes are only caused by drift. Effective population sizes were determined for the prefarm‐reared tadpole supplementation phase (1997–2005), the postfarm‐reared tadpole supplementation phase (2005–2014), and the entire sample period (1997–2014).

### *N*_b_/*N*_c_ and *N*_e_/*N*_c_ estimates

Census size (*N*_c_) values were estimated from demographic data based on the number of adults captured during each breeding season (Richter and Seigel 1996, 1997, 1998; Seigel and Kennedy 2000; Seigel et al. 2001; Thurgate et al. 2002; Sisson 2003, 2004, 2005; Baxley and Qualls 2006, 2007; Sisson et al. 2008; Lee 2011; J. Tupy, pers. comm.). Effective size to census size ratios were calculated for estimates derived from both the single‐sample and temporal estimators. Ratios were calculated via the discrete generation method suggested by Waples (2005), using the harmonic means of the census size estimates from the sample proceeding the sampling period (*N*_(−1)_). However, amphibians have overlapping generations, and recognized bias and inaccuracies could be introduced into these estimates.

## Results

### Genetic variation and structure by year

**Figure 1:**
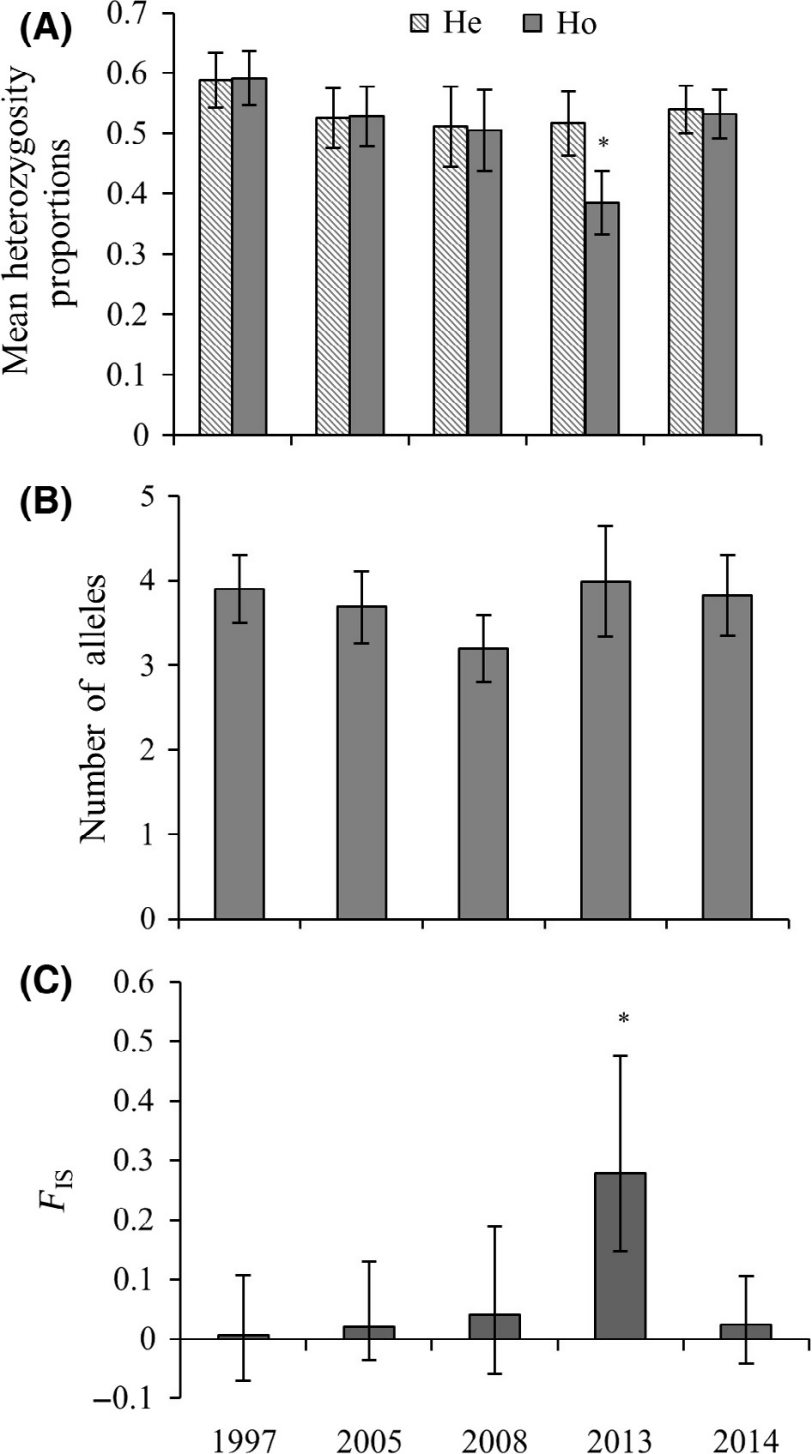
Mean ± 1 SE observed and expected heterozygosities (A), allelic richness using rarefaction (B), Wright's inbreeding coefficient, *F*_IS_ (C) values across nine microsatellite loci for *Lithobates sevosus* 1997–2014. Asterisks (*) indicate significance between pairwise comparisons. Rarefaction number by year = 17 individuals.

**Figure 2:**
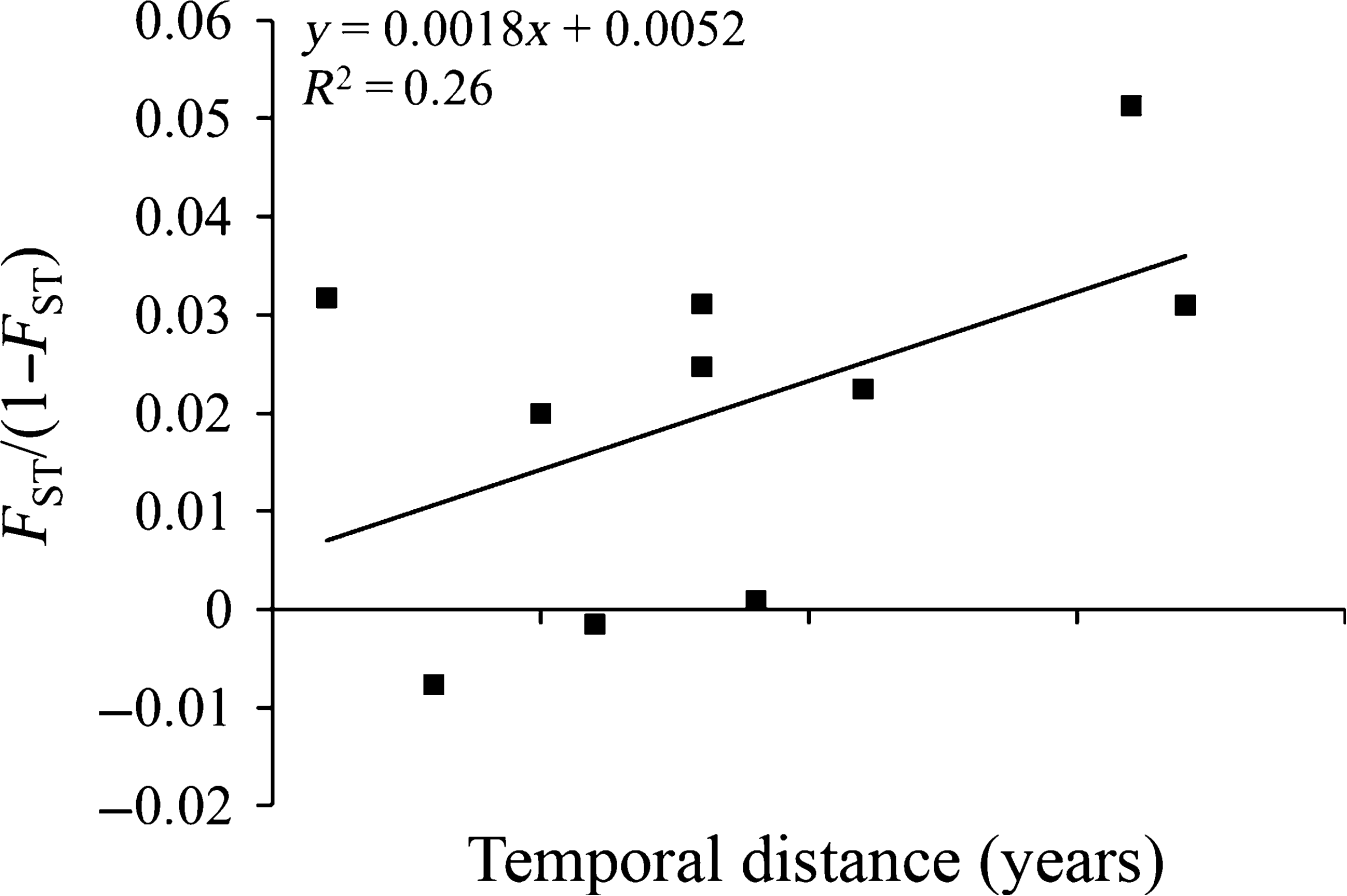
Isolation by temporal distance for pairwise *F*_ST_ comparisons for *Lithobates sevosus* from 1997 to 2014 based on nine microsatellite loci.

Null alleles were suggested to be present in 3 years of the sampling period. Data were reanalyzed upon removal of loci with null alleles, and the results were similar. Additionally, there was no consistent pattern across years or loci, so all loci were included in final analyses. Deviations from Hardy–Weinberg equilibrium occurred at locus Lica 41 in the year 2013. No pairs of loci exhibited linkage disequilibrium. When analyzing the data year‐by‐year the amount of genetic variation within *L. sevosus* remained relatively stable throughout the sample period, with the exception of year 2013. Observed and expected heterozygosity ranged from 0.39 to 0.59 and 0.51 to 0.59, respectively, with significant differences occurring in observed heterozygosity (Fig. 1A) (observed heterozygosity: $\chi_{4}^{2}$ = 13.42, *P* = 0.01; expected heterozygosity: $\chi_{4}^{2}$ = 4.90, *P* = 0.30). The Wilcoxon signed‐rank test indicated a significant pairwise difference in observed heterozygosity values for 2013 compared to all other years, with uncorrected *P*‐values ranging 0.01–0.02. Allelic richness ranged from 3.20 to 3.99, with a nonsignificantly decreasing, then increasing over the sample period (Fig. 1B) ($\chi_{4}^{2}$ = 2.42, *P* = 0.66). Wright's inbreeding coefficient values ranged from 0.01 to 0.28, with significant differences over the sample period (Fig. 1C) ($\chi_{4}^{2}$ = 10.04, *P* = 0.04). Sample year 2013 was different from all years except 2008, with *P*‐values ranging 0.02–0.04. A heterozygote deficiency was detected in 2013, with *F*_IS_ values significantly >0 (95% CI 0.08–0.41). The overall *F*_ST_ value was 0.023 (95% CI 0.01–0.04), indicating weak genetic differentiation among years. Significant differentiation existed between all pairs of sample years, with the exception of years 2008 versus 2014, 2005 versus 2008, and 2005 versus 2014 (Table 1). Pairwise *F*_ST_ comparisons exhibited a positive relationship—indicating that approximately one‐fourth of the variation in genetic distance can be attributed to temporal distance (Fig. 2) (Mantel test with 10,000 permutations, *R* ^2^ = 0.26, *P* = 0.13).

**Table 1:** Pairwise *F*_ST_ values for *Lithobates sevosus* from 1997 to 2014 based on nine microsatellite loci

	2014	2013	2008	2005	1997
2014	–	0.03*	0	0.001	0.03*
2013		–	0.02*	0.02*	0.05*
2008			–	0	0.02*
2005				–	0.03*
1997					–

Pairwise *F*_ST_ values calculated using FSTAT version 2.9.3.2 (Goudet 1995) with asterisks indicating significance at *P* < 0.05 calculated using Arlequin version 3.5.2.1 (Excoffier and Lischer 2010).

### Genetic variation by conservation period and stage

**Figure 3:**
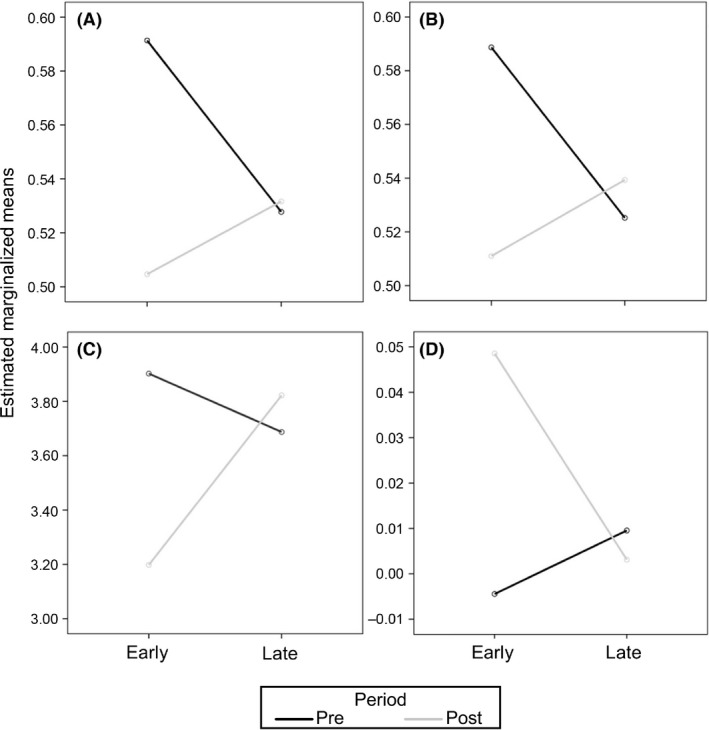
Estimated marginal means from two‐way repeated measures ANOVA for observed heterozygosity (A), expected heterozygosity (B), allelic richness using rarefaction (C), and Wright's inbreeding coefficient (D) for *Lithobates sevosus* with year 1997 representing “pre” and “early”, year 2005 representing “pre” and “late”, year 2008 representing “post” and “early”, and year 2014 representing “post” and “late” conservation efforts.

No statistical relationship was found between conservation period or stage on heterozygosity, allelic richness, or inbreeding. Graphically, however, a nonsignificant antagonistic interaction is revealed between stage x period and heterozygosity, allelic richness, and inbreeding (Fig. 3) (observed heterozygosity: *F*_1_ = 3.03, *P* = 0.12; expected heterozygosity: *F*_1_ = 5.02, *P* = 0.06; allelic richness: *F*_1_ = 2.16, *P* = 0.18; Wright's inbreeding coefficient: *F*_1_ = 0.42, *P* = 0.54).

### *N*_b_ and *N*_e_ estimates

The Bayesian partial likelihood method of Tallmon et al. (2008) produced *N*_b_ estimates ranging from 30.5 to 58.6 over the sample period, with 2005 containing the smallest value and 2014 containing the largest (Table 2). The analyses for this method omitted loci RsevC02 and RsevF01 due to missing data. The pseudolikelihood method of Wang (2001) produced *N*_e_ estimates that increased over the study period—with greater values in the postheadstarting period (2005–2014) than in the preheadstarting period (1997–2014) (Table 3). To test for the possible effects of excluding loci RsevC02 and RsevF01 on *N*_b_ and *N*_e_ estimates, the pseudolikelihood method was rerun also omitting these loci. The results produced *N*_e_ estimates around 1.1 times smaller than those with nine loci. Because of this minute difference, further interpretation proceeded using nine loci for the pseudolikelihood method.

**Table 2:** Effective number of breeders estimates with 95% confidence intervals via a Bayesian partial likelihood single‐sample method for *Lithobates sevosus* based on nine microsatellite loci

Year	Tallmon et al. (2008)	*N*_b_/*N*_c_
1997	45.5 (31.4–54.9)	0.48
2005	30.5 (23.7–66.0)	0.53
2014	58.6 (43.0–144.0)	0.77

**Table 3:** Effective population size estimates with 95% confidence intervals via a pseudolikelihood temporal method for *Lithobates sevosus* based on nine microsatellite loci

Year	Wang (2001)	*N*_e_/*N*_c_
1997–2005	25.54 (13.87–62.64)	0.37
2005–2014	46.63 (22.27–166.19)	0.71
1997–2014	32.99 (21.06–53.98)	0.48

### *N*_b_/*N*_c_ and *N*_e_/*N*_c_ estimates

The estimates from the Bayesian partial likelihood analysis and the pseudolikelihood method were used to calculate the *N*_b_/*N*_c_ and *N*_e_/*N*_c_ ratios, respectively. *N*_b_/*N*_c_ estimates ranged from 0.48 to 0.77, with estimates increasing with time (Table 2). *N*_e_/*N*_c_ estimates ranged from 0.37 to 0.71 and exhibit the same trend of increasing with time (Table 3).

## Discussion

### Genetic variation and structure by year

Population isolation and small population size are known drivers for reduction in genetic variation (Frankham 1996). Previous research found reduced levels of genetic variation in *L. sevosus* (Richter et al. 2009). The findings of this study are consistent with Richter et al. (2009) and suggest that these reduced levels have remained generally stable and appear to be improving over the sample period 1997–2014.

In terms of trends in genetic variation, both levels of observed and expected heterozygosity decreased and then increased during the sample period. This trend could be attributed to the implementation of various management practices aimed at rescuing *L. sevosus* from extinction. Since 2002, a subset of eggs deposited in Glen's Pond have been transferred to cattle tanks and reared until metamorphosis for release into the population (Thurgate et al. 2002). These efforts have likely assisted in maintaining the levels of genetic variation and preventing further genetic erosion. Additionally, allelic richness declined and then rebounded following year 2008. Previous research documented a reduction in allelic diversity within *L. sevosus*, and our current data provide new evidence for the regaining of alleles, which indicates, in terms of mutation‐drift equilibrium, fewer alleles are being lost by genetic drift (Frankham et al. 2004). This phenomenon is likely an indication of the success of management practices. Alleles are the raw material of evolution and provide the variation for which selection acts on; therefore, the presence of more alleles could indicate an increased evolutionary potential (Allendorf 1986).

Wright's inbreeding coefficient (*F*_IS_) within *L. sevosus* followed an increasing then decreasing pattern over the sample period. This trend can be strengthened by demographic data, which reveals an inverse pattern of a decreasing then increasing number of breeding individuals (Richter and Seigel 1996, 1997, 1998; Seigel and Kennedy 2000; Seigel et al. 2001; Thurgate et al. 2002; Sisson 2003, 2004, 2005; Baxley and Qualls 2006, 2007; Sisson et al. 2008; Lee 2011; J. Tupy, pers. comm.). This relationship underscores the importance of using demographic data to bolster genetic data.

The highest *F*_IS_ value occurred in 2013. These values represent the probability that two alleles are identical by descent—with a value of 0.278 in 2013 at the level expected of full‐sib (brother x sister) mating (Wright 1921). The cause for this large amount of inbreeding in 2013 followed by an immediate reduction in 2014 is unknown, but likely due to a suite of factors pertaining to the stochastic nature of *L. sevosus*' highly variable reproductive success and output (Richter et al. 2003). First, a low reproductive effort (31 egg masses) could cause a drop in genetic diversity by having only a subset of the population represented. Additionally, few adults breed more than once, with an average rate of return between 16 and 22% (Richter and Seigel 2002). Therefore, each breeding season is largely dependent on new breeders, who reach sexual maturity between 1 and 3 years of age (Richter and Seigel 2002). This is important to note because only a small breeding event occurred in 2011 due to drought, and no tadpoles were available for headstarting or translocation (Lee 2011). The substantial drop in genetic diversity and increase inbreeding in year 2013 could be a delayed effect of no population recruitment in 2011. Year 2013 therefore serves as a reminder of the effects of reproductive and environmental stochasticity on genetic variation of isolated populations.

Richter and Nunziata (2014) found that inbreeding depression might be more likely to be expressed in some years and not others, with this differential expression being largely dependent on variable environmental conditions and intensity of natural selection. Our estimates of inbreeding in *L. sevosus* are in line with these findings. Richter and Nunziata (2014) found evidence of inbreeding depression within 1997. This year had the lowest *F*_IS_ level of our sample period; therefore, we can speculate that if inbreeding depression is present in the year with the lowest *F*_IS_ values, then inbreeding depression may also be present, perhaps in larger amounts, in the other years, depending on environmental conditions. However, it is important to note that Richter and Nunziata (2014) found *F*_IS_ values to decrease from eggs to metamorphosed individuals, with less inbred individuals having a greater survival to metamorphosis. Because our tissue samples are from eggs, the *F*_IS_ levels found may be larger than what is present within the adult population (Ficetola et al. 2011; Richter and Nunziata 2014). Therefore, interpreting our data in the context of this study is complicated due to our tissue type and because of the implementation of conservation management practices augmenting the number of metamorphs.

Additionally, although inbreeding can lead to inbreeding depression, which results in deleterious consequences like reduced fitness, sometimes inbreeding can also be beneficial. For example, inbreeding increases levels of homozygosity, which then exposes deleterious alleles to selection. This purging of alleles occurs more rapidly than it otherwise would under the normal conditions of random mating. This, in turn, acts to reduce the magnitude of inbreeding depression (Frankham et al. 2004). Purging selection against deleterious mutants has been previously documented in *L. sevosus* and other isolated populations of anurans, with a decrease in sublethal homozygous genotypes from larvae to metamorphosis (Ficetola et al. 2011; Richter and Nunziata 2014). Therefore, although *L. sevosus*' small population size and history of inbreeding is cause for concern, the purging of alleles could play a positive role in preventing this species from entering an extinction vortex.

Temporal stability in population structure is not often measured due to the large amount of data required; however, there is an acknowledged need for these estimates (Hoffman et al. 2004). Temporal stability in genetic structure appears to be dependent on a suite of factors such as environmental stochasticity, immigration, and genetic drift—with multiple studies documenting both temporal stability in genetic structure (Bernal‐Ramírez et al. 2003; Hoffman et al. 2004; Eldridge et al. 2009; Cuveliers et al. 2011; DeFaveri and Merilä 2015) and temporal instability (Crispo and Chapman 2010; Norén et al. 2011; Kesäniemi et al. 2014; Holmes 2015). Our findings suggest a weak genetic structuring over time in *L. sevosus*. This weak temporal structuring is likely the result of random genetic drift and inbreeding caused by nonrandom mating due to small population size (Frankham et al. 2004).

### Genetic variation by conservation period and stage

The graphical interaction between conservation period (pre or post) and conservation stage (early or late) reveals that the conservation practices have had a positive effect on heterozygosity, allelic richness, and inbreeding. Both observed and expected heterozygosity and allelic richness show values decreasing from early to late stages in the preconservation period, and then increasing from early to late stages in the postconservation period. Similarly, inbreeding values increase from early to late stages in the preconservation period and then decrease from early to late stages in the postconservation period. These slope changes suggest that the management practices in place have reversed the previous trajectory of decreasing genetic variation and increasing inbreeding and have replaced it with a more promising outlook.

### *N*_b_ and *N*_e_ estimates

Effective population size is an important parameter that influences population viability and conservation management decisions. This estimate can be interpreted both at the global and the local scale, depending on the scope of the project. At the global scale, information such as long‐term viability, adaptive potential, and maintenance of genetic variation can be gained. At the local scale, immediate threats to population persistence can be revealed (Luikart et al. 2010). Because *L. sevosus* exists as one population, *N*_e_ (or *N*_b_) estimates can be interpreted at both scales.

In general, the single‐sample *N*_b_ and temporal *N*_e_ methods produced comparable results, with values increasing temporally. The increase over time in effective size coincided with the implementation of conservation efforts, especially the headstarting program. This trend suggests that the conservation management practices in place for *L. sevosus* are effective at both conserving and increasing effective population size. Specifically, the estimates produced by the single‐sample Bayesian partial likelihood method are similar to demographic data collected over the sample period. During the middle of the sample period, there were successive years with little to no reproductive output because of drought and an emergent disease—a *Perkinsus*‐like pathogen that caused major mortalities in 2003 (Overstreet and Lotz 2004). Following the initiation of headstarting to circumvent pond drying and disease outbreaks, reproductive output increased (Richter et al. 2003; Thurgate et al. 2002; Sisson 2003, 2004, 2005; Baxley and Qualls 2006, 2007; Sisson et al. 2008; Lee 2011; J. Tupy, pers. comm.; J. H. K. Pechmann, unpublished data). The single‐sample Bayesian partial likelihood method estimates follow the same decreasing and then increasing trend, bolstering our confidence in the accuracy of this estimate. Further, the increase in *N*_b_ and *N*_e_ estimates with time further demonstrates that the reduction in genetic diversity in year 2013 does not appear to be affecting the number of genetically contributing individuals each breeding season.

### *N*_b_/*N*_c_ and *N*_e_/*N*_c_ estimates

Frankham (1995), in a review encompassing 102 species including most major taxa (i.e., insects, mollusks, amphibians, reptiles, birds, mammals, and plants), cites three main factors influencing effective size to census size ratio estimates: fluctuations in population size, variance in individual reproductive success, and unequal sex ratios. There is no consensus on what is “normal” for natural populations, with Frankham (1995) suggesting ratios of around 0.11, while Nunney (1993) proposed through theoretical analyses that the interplay of mating system and generation time gives values around 0.5. Specific to anurans, Hoffman et al. (2004) estimated *N*_e_/*N* ratios of at least 0.10 and indicated that this ratio is probably higher. However, few additional studies have examined what is a typical ratio for anurans, if these ratios vary for different groups of anurans, and if ratios are temporally stable. Our estimates (0.369–0.771) are in line with the theoretical values of Nunney (1993). Jehle et al. (2005) found similar, large *N*_e_/*N*_c_ ratios in amphibians (specifically newts) with small population sizes. Similarly, species of conservation concern tend to have larger *N*_e_/*N*_c_ (or *N*_b_/*N*_c_) ratios than stable populations (Palstra and Ruzzante 2008; Saarinen et al. 2010). This phenomenon was first described by Ardren and Kapuscinski (2003) as genetic compensation—an increase in *N*_e_/*N*_c_ (or *N*_b_/*N*_c_) ratios at low population sizes. Genetic compensation suggests that when population sizes are low, most individuals participate in breeding activities. In an evolutionary context, this compensation would lessen genetic variation reduction in times with low population numbers (i.e., bottlenecks) by reducing inbreeding. These results suggest that *L. sevosus* might be experiencing genetic compensation in concert with effective conservation management practices that have stabilized *N*_e_/*N*_c_ (and *N*_b_/*N*_c_) ratios and genetic variation over the sample period.

### Current status

In the 2015 breeding season, 92 egg masses were laid in Glen's Pond—a greater amount than any year in our study (J. Tupy, pers. comm.). Also, 18 egg masses were laid in Pony Ranch Pond via natural migration and recruitment. This large reproductive output is consistent with the trend seen in previous years, which show an increasing amount of egg masses laid following the implementation of conservation management practices. The findings of this study support and strengthen the large demographic dataset, and show that as reproductive output continues to increase, so does genetic variation and effective size.

## Conclusions

Overall, this study illustrates three major findings. First, the current management practices in place for *L. sevosus* appear to have been successful at preventing further genetic erosion. The addition of headstarting tadpoles appears to be an effective way of maintaining genetic variation in study systems subject to variable reproductive output due to environmental stochasticity or disease. However, further efforts are still needed to continue restoring genetic variation to higher levels. Second, *L. sevosus* exhibits weak temporal genetic structuring. This differentiation is likely due to increased effects of genetic drift and inbreeding in small, isolated populations. Future studies should continue to focus on temporal genetic structuring, as it provides genetic information on microevolutionary changes. Third, the effective population size and effective number of breeders estimates can be variable, but overall, effective population size of *L. sevosus* is around half that of the estimated census size. These *N*_e_/*N*_c_ and *N*_b_/*N*_c_ estimates contribute to the literature on anuran effective population size—helping to aid in the search for a common ratio among taxa, and suggest that genetic compensation might be acting within this system, reducing the loss of genetic diversity. Temporal estimates demonstrated that *N*_e_ increased, potentially because of headstarting efforts, indicating further success of the conservation plans and that the decrease in heterozygosity in year 2013 does not appear to be affecting the number of genetically contributing individuals each breeding season. Despite the success of multiple management practices in place, *L. sevosus* still has a reduced amount of genetic variation, and new, interconnected populations must be established in order to improve population persistence and ensure future viability of the species. The recent breeding events at Pony Ranch Pond provide encouraging possibilities for the establishment of population connectivity. Additional wetland restorations could benefit from the use of head‐started tadpoles from Glen's Pond and captive zoo populations, although careful consideration must be taken to eliminate the risk of transferring pathogens from captive to natural populations and between natural populations.

## Conflict of Interest

None declared.
